# Learning to Predict Crystal Plasticity at the Nanoscale: Deep Residual Networks and Size Effects in Uniaxial Compression Discrete Dislocation Simulations

**DOI:** 10.1038/s41598-020-65157-z

**Published:** 2020-05-19

**Authors:** Zijiang Yang, Stefanos Papanikolaou, Andrew C. E. Reid, Wei-keng Liao, Alok N. Choudhary, Carelyn Campbell, Ankit Agrawal

**Affiliations:** 1 Department of Electrical and Computer Engineering, Northwestern University, Evanston, USA; 2 Department of Mechanical & Aerospace Engineering, West Virginia University, Morgantown, USA; 3 Center for Theoretical and Computational Materials Science, National Institute of Standards and Technology, Gaithersburg, USA

**Keywords:** Computational methods, Characterization and analytical techniques

## Abstract

The density and configurational changes of crystal dislocations during plastic deformation influence the mechanical properties of materials. These influences have become clearest in nanoscale experiments, in terms of strength, hardness and work hardening size effects in small volumes. The mechanical characterization of a model crystal may be cast as an inverse problem of deducing the defect population characteristics (density, correlations) in small volumes from the mechanical behavior. In this work, we demonstrate how a deep residual network can be used to deduce the dislocation characteristics of a sample of interest using only its surface strain profiles at small deformations, and then statistically predict the mechanical response of size-affected samples at larger deformations. As a testbed of our approach, we utilize high-throughput discrete dislocation simulations for systems of widths that range from nano- to micro- meters. We show that the proposed deep learning model significantly outperforms a traditional machine learning model, as well as accurately produces statistical predictions of the size effects in samples of various widths. By visualizing the filters in convolutional layers and saliency maps, we find that the proposed model is able to learn the significant features of sample strain profiles.

## Introduction

Prediction of mechanical behavior up to failure is commonly achieved using constitutive laws written as equations in terms of phenomenological parameters in the cases where physical laws are not clear or for the purpose of simplification. The parameters are obtained experimentally (at the manufacturing stage) for individual classes of materials, thus classified by composition, prior processing and load history, all of which affect the material’s micro-structure and thus its behavior^1^. Beyond processing routes, materials have been known to also be highly sensitive to micro-structure changes, especially when used at extreme conditions such as small volume, high temperature, high pressure, and high strain rates^2–11^. Such extreme conditions are experienced in numerous applications at the technological and industrial frontiers. Thus, constitutive laws are difficult to apply when the micro-structural changes brought by operating conditions are unknown. In order to assess the yield and failure strength values, current practice requires non-destructive characterization methods at the nanoscale that can swiftly assess mechanical properties. The case study in this work represents possibly one of the most challenging, but benchmarked^12^, applications of micromechanics. More specifically, we investigate, using synthetic data from discrete dislocation plasticity simulations^12–15^ how such non-destructive characterization can effectively work in the realistic scenario of assessing and predicting the strength of small finite volumes by using digital image correlation (DIC)^16^ techniques.

Changes in the dislocation structure of a material caused by prior processing of a crystal may not be evident from a visual inspection of a sample surface (especially if polished), but the material properties may be dramatically influenced after heavy prior plastic deformation, without a change of the chemical composition^17^. A step forward towards non-destructive characterization can be achieved through detailed quantitative surface deformation data, that have recently become readily accessible through DIC^16^. In addition, recently developed data-driven methods have been utilized to identify initial strain deformation level of synthetic samples of small finite volumes, produced through discrete dislocation dynamics (DDD) simulations. Papanikolaou *et al*.^13^ emulated DIC using DDD, by simulating thin film uniaxial compression of samples under different states of prior deformation, and removing the average residual plastic distortion before reloading. It is worth noting that the utilized DDD model was benchmarked to minimally model size effects in small finite volumes^12^. In^13^, two-point strain correlations at different locations were used to capture spatial features of dislocations, through their resulting strain profiles. In that work, dislocation classification was performed using Principal Component Analysis (PCA)^18^ and continuous k-nearest neighbors clustering algorithms^19^. Analogous classifications of dislocation structures have since been extended in disordered dislocation environments^20^ and three dimensional DDD samples^21^, as well as continuous plasticity models^22^ using a variety of data science approaches. However, the problems in existing works are either in simpler models of dislocation dynamics^20^ or with less challenging limits of the model (i.e. large sample widths)^13^. Thus, it has yet to become clear how to practically, accurately and efficiently assess, using machine learning, dislocation plasticity features in realistic scenarios that can be tested experimentally. Here, we show that deep learning is a key component towards practical, accurate and efficient non-destructive characterization of dislocation plasticity.

Deep learning has recently become an immensely popular research area in machine learning, and it has led to groundbreaking advances in various fields such as object recognition^23–25^, image segmentation^26–28^ and machine translation^29–31^. The success of deep learning has also motivated its application in other scientific fields, such as healthcare^32–34^, chemistry^35–37^ and materials science^38–47^. In^38^, 3D convolutional neural network was developed to model homogenization linkages for high-contrast two-phase composite material system. In^39^, generative adversarial networks and Bayesian optimization were implemented for material microstructural design. Ryczko *et al*.^40^ propose a convolutional neural network for calculating the total energy of atomic systems. In^36^, recursive neural network approaches are applied to solve the problem of predicting molecular properties. Due to the high learning capability and model generalization, deep learning could be a promising technique for crystal plasticity research.

**Figure 1:**
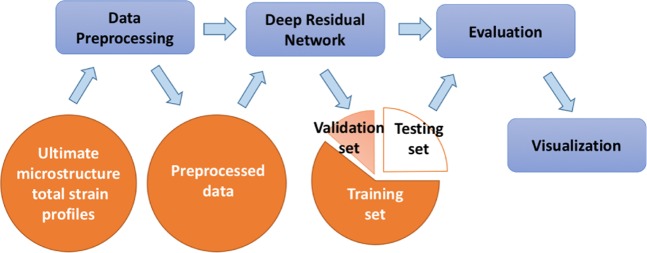
The flowchart of the proposed method.

For providing accurate predictions in an efficient manner, we propose a deep residual network to predict the strength and work hardening features of a sample from a given set of strain images that are acquired at small-deformation (≤1%) testing. More specifically, the flowchart of this work is presented in Fig. 1. We use synthetic datasets that are generated by DDD simulations of uniaxial compression in small finite volumes of a metal with elastic properties reminiscent of single crystal Al and a range of sample widths *w* that extend from 62.5 nm to 2 *μ*m (see Table 2). The effect of the potentially varying loading orientation is emulated by using two models, with activation of either one (+30° with respect to loading axis) or two slip systems (±30°) (labeled as one-slip or two-slip)^12–15^. For each width, datasets may also be characterized by low, medium or high initial prior strain deformation levels (low:0.1%, medium:1.0% and high:10.0% prior strain respectively), as well as small or large characterization strain (labeled as Small-reload for 0.1% or Large-reload for 1% reloading strain). For each case of a *w*, loading orientation, prior strain and reloading strain, the sample strength and plastic flow varies significantly, so we produce 20 simulations of distinct initial conditions^13^. As found in^12^, sample strength displays a strong size effect *σ*_*y*_ ~ *w*^−0.55^, while plastic flow becomes significantly noisier at smaller *w*, but also shows strong dependence on all varied parameters.

In the predictive model we propose here, a data preprocessing step is applied to augment the dataset and convert the strain profile to the format that is suitable for the proposed model. The preprocessed data is split into a training set, a validation set and a testing set. The training set and validation set are used to train the proposed model, while the testing set is used to evaluate its performance. The proposed model is compared to a benchmark method that computes the two-point correlation of strain profiles, then applies PCA to get the reduced-order representations, and finally fits a prediction model. The experimental results show that the deep learning approach can significantly improve the classification accuracy by up to 35.17% and thus accurately identify the initial strain deformation level of the samples. Moreover, the classification accuracy is further improved by the ensemble of differently trained deep residual networks. By using feature maps of the second to last convolutional layer to represent the input strain profiles, we can define a distance between two strain profiles, and subsequently identify the nearest neighbors for the test samples within the training set. We find that this process can provide us with an accurate statistical prediction of the stress-strain curves for the test samples not seen during training. In addition, by visualizing the filters in the convolutional layer and saliency maps using the proposed model, we show that the proposed model can successfully capture the significant information and salient regions from the strain profiles. Most importantly, unlike previous work^20^ using physically motivated features as inputs, our proposed method only takes raw strain profiles as inputs without any ad hoc assumptions about the material, and it has been evaluated under multi-slip conditions. Thus the proposed method could provide a more robust predictive model and be easily extended to other material systems.

## Results

The performance of the proposed model is evaluated in section 2.1, and the proposed model is used to predict the stress-strain curve for a sample of interest in section 2.2. Filters in convolutional layers and saliency maps of the proposed model are visualized in section 2.3.

### Prediction of initial strain deformation level

In this section, the performance of the proposed model is compared with a benchmark method, which is the correlation function based method. The correlation function based method is widely used in materials science research^48–50^. For the correlation function based method, two-point correlation function of the strain profile is first computed, then Principal Component Analysis is applied to obtain the reduced-order representations, and finally Random forest^51^ is implemented to train the predictive model.

**Figure 2:**
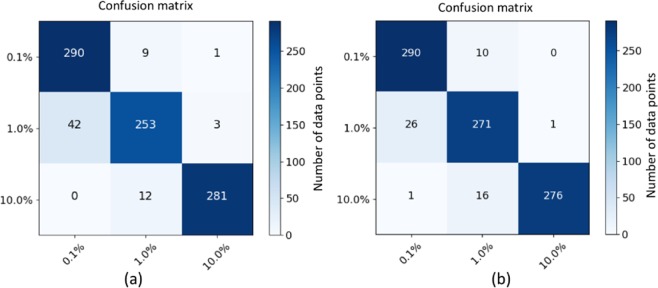
The confusion matrix of the proposed CNN (**a**) and ensemble of three deep learning models (**b**).

Table 1 shows the results of the correlation function based method and the proposed model. We can observe that correlation function based method can get 68.24% classification accuracy, while the proposed model can achieve a significantly better 92.48% classification accuracy. In addition, Fig. 2(a) shows the confusion matrix of the proposed model. It shows that low initial strain deformation level (i.e., 0.1% strain class) and high initial strain deformation level (i.e., 10.0% strain class) can be accurately predicted, which only have 10 and 12 misclassified samples, respectively. Medium initial strain deformation level (i.e., 1.0% strain class) has relatively worse predictions where 42 samples and 3 samples are misclassified as 0.1% and 10.0% strain classes, respectively.

**Table 1:** Prediction accuracy of different models.

Architecture	Accuracy
Correlation function based method	68.24%
*input* − *conv16* − *pool* − (*Res*32 × 3) × 2 − *pool* − (*Res*64 × 3) × 2 − *pool* − *avgpool* − *output* **(The proposed CNN)**	92.48%
*input* − *conv16* − *pool* − *Res*32 × 4 − *pool* − *Res*64 × 4 − *pool* − *avgpool* − *output* with *L*2 regularization	91.58%
*input* − *conv16* − *pool* − *Res*32 × 4 − *pool* − *Res*64 × 4 − *pool* − *avgpool* − *output* without *L*2 regularization	90.01%
Ensemble of above three deep learning models	**93.94%**

**Figure 3:**
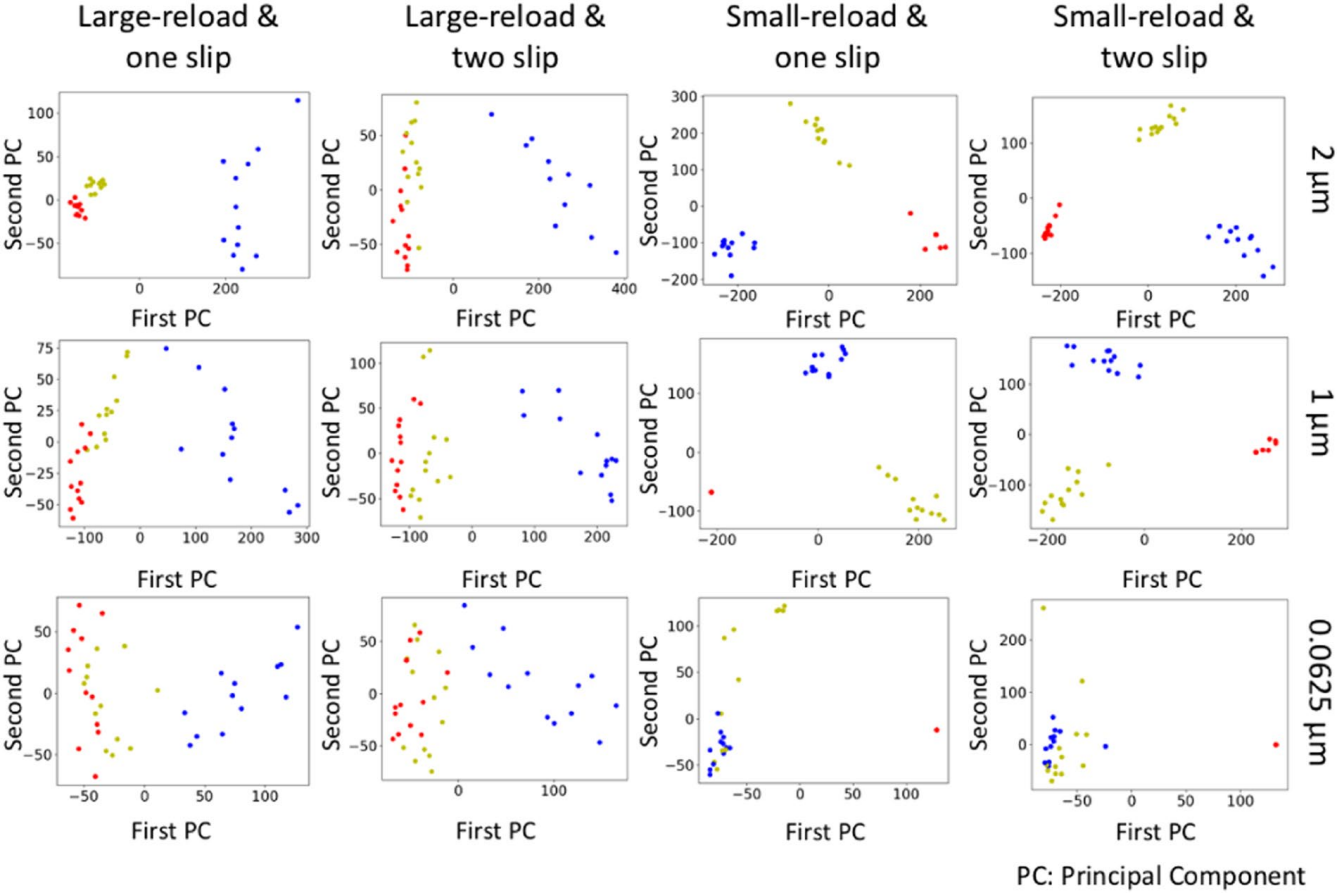
Projection of each subset data on first two principal components of the outputs of the global average pooling layer of the proposed model. 0.1%, 1.0% and 10.0% initial strain deformation levels are represented by red color, yellow color and blue color, respectively. Each row from top to bottom presents the plots of samples with the width of 2 *μm*, 1 *μm* and 0.0625 *μm*, respectively. Each column from left to right shows the plots of samples of a combination of slip type and reload strain condition, which are large-reload & one slip, large-reload & two slip, small-reload & one slip, and small-reload & two slip, respectively.

Intuitively, the smaller the sample width is, the harder the prediction should be. Table 2 presents the classification accuracy of samples with different sample widths. We can observe that when sample width is large (i.e., 2 *μm* and 1 *μm*), model’s performance is the best and both accuracies are above 95%. As the sample width decreases, the classification accuracy also decreases. The accuracies are around 89% for 0.125 *μm* and 0.0625 *μm*, which agrees with our intuition. More specifically, the classification accuracy for each subset data is listed in Table 2. We also extract the outputs of the global average pooling layer of the proposed model, and project the outputs on the first two principal components in Fig. 3. The results show that the proposed model performs better when samples have a large width (e.g., 2 *μm* and 1 *μm*) with small-reload, and the corresponding clusters of three strain classes are clearly separated in Fig. 3. On the other hand, the classification accuracy decreases for samples with a small width (e.g., 0.0625 *μm*) and large-reload where the clusters of 0.1% and 1.0% strain classes are significantly overlapped in Fig. 3.

**Table 2:** Prediction accuracy of the proposed CNN and ensemble of three deep learning models.

Prediction accuracy of the proposed CNN						
Width (*μm*)	2	1	0.5	0.25	0.125	0.0625
Total Accuracy	95.24%	96.00%	93.15%	92.67%	88.51%	89.33%
Small-reload & two slip	100%	100%	94.59%	100%	92.11%	92.31%
Small-reload & one slip	97.22%	100%	97.22%	94.44%	100%	94.44%
Large-reload & two slip	89.19%	89.74%	94.59%	89.74%	78.95%	79.49%
Large-reload & one slip	94.44%	94.44%	86.11%	86.11%	83.33%	91.67%
**Prediction accuracy of ensemble of three deep learning models**						
Width (*μm*)	2	1	0.5	0.25	0.125	0.0625
Total Accuracy	97.28%	97.33%	95.21%	94.00%	89.19%	90.67%
Small-reload & two slip	100%	100%	91.89%	97.44%	89.47%	94.87%
Small-reload & one slip	100%	100%	94.44%	91.67%	100%	94.44%
Large-reload & two slip	89.19%	92.31%	97.30%	92.31%	84.21%	82.05%
Large-reload & one slip	100%	97.22%	97.22%	94.44%	83.33%	91.67%

Szegedy *et al*.^52^ show that an ensemble of different trained deep learning models can be used to further improve model’s performance. Thus in this work, we also use an ensemble of three trained deep learning models whose classification accuracies are above 90%. Table 1 shows the architectures and classification accuracy of the proposed model and the other two deep learning models (see section 4.5 for detailed information about deep learning models). The second and third models have the same architecture, and the difference is that one uses *L*2 regularization with penalty factor as 0.0001 for all the convolutional layers, while the other does not use *L*2 regularization. Note that other parameter settings, such as activation functions, optimizer and early stopping, are the same as the proposed model. Meanwhile, because the dataset is relatively small to train a deep learning model, data preprocessing approach is used to augment the dataset (see section 4.4 for detailed information about data preprocessing). Particularly, image cropping is used so that each strain profile ends up with 24 crops. Therefore, the final probabilities of a sample are averaged over its crops and over all the three deep learning models, and the final prediction is the class with the highest probability. Particularly, the probability is calculated by softmax function, which is a normalized exponential function. It takes a vector of *K* real numbers as input, and normalizes it into a probability distribution consisting of *K* probabilities proportional to the exponentials of the input numbers. Using an ensemble of three deep learning models, the classification accuracy is improved to 93.94% as shown in Table 1. The confusion matrix in Fig. 2(b) shows that fewer samples are misclassified as low initial strain deformation level (i.e., 0.1% strain class) from medium initial strain deformation level (i.e., 1.0% strain class) compared with the single proposed model, while the number of misclassified samples for the other two initial strain deformation levels are similar to the single proposed model.

Moreover, Table 2 shows that the classification accuracy is improved by around 1% for all the sample widths, and the trend still holds that the accuracy decreases with decreasing sample width. Particularly, the classification accuracy for almost all the sample widths with large-reload is significantly improved compared to the single proposed model. In addition, the worst classification accuracy is improved from 78.95% (i.e., accuracy for samples with 0.125 *μm* width, large-reload and two slip in single proposed model) to 82.05% (i.e., accuracy for samples with 0.0625 *μm* width, large-reload and two slip in ensemble model).

### Prediction of the stress-strain curve

**Figure 4:**
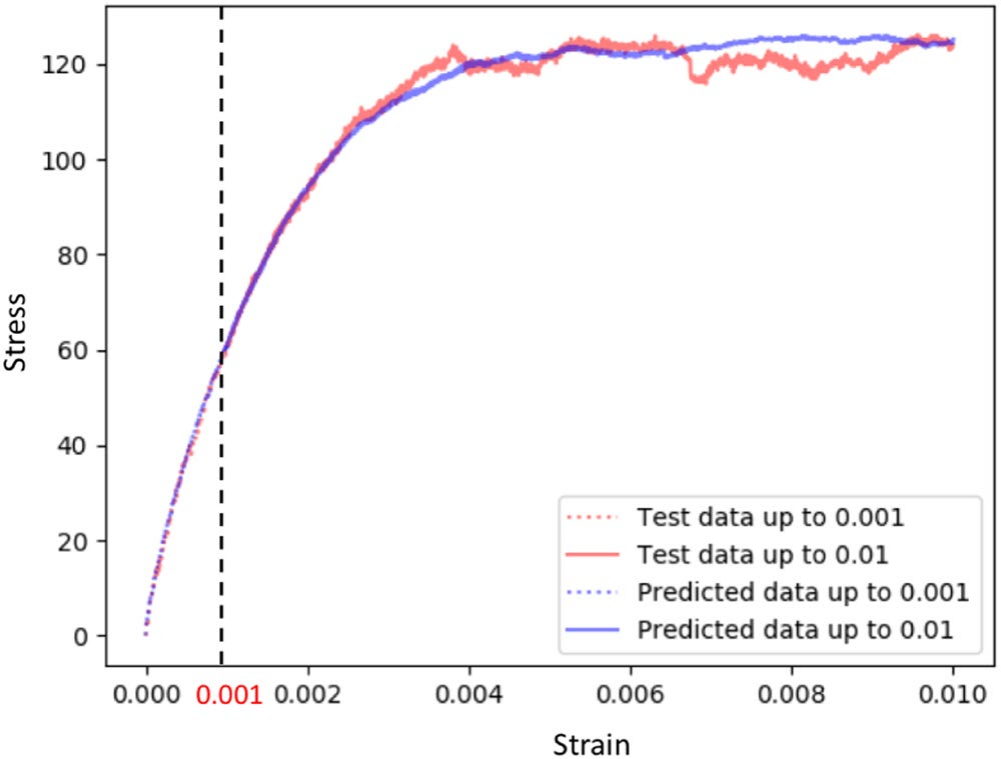
An example of true and predicted stress-strain curves of a strain profile. The red dash and solid curves show the true stress strain curves of the strain profile, respectively. The blue dash and solid curves show the predicted stress strain curves of the strain profile, respectively.

Another important challenge is to predict the stress-strain curve for a sample of interest. More specifically, given the initial deformation strain, it is straightforward to know the dislocation density of the crystal, which provides information for predicting the strength of the crystal. In other words, if a model can accurately predict the initial deformation strain, it can be used to predict the stress-strain curve for a sample of interest. Figure 4 shows an example of true and predicted stress-strain curves of a given strain profile. More specifically, given a stress-strain curve up to (only) 0.1% reloading strain of a testing strain profile (i.e., the red dash curve), the stress-strain curve up to 1% reloading strain (i.e., red solid curve) could be predicted with the proposed model. In Fig. 4, the predicted stress-strain curves up to 0.1% and 1% reloading strain are shown as blue dash and solid curves, respectively, which are the averaged stress-strain curves of “neighbors” of the testing strain profile calculated using the proposed model.

To achieve this, we use the outputs of second to last layer of the proposed model as image features to represent input images. For each strain profile with small-reload (i.e., 0.1% reloading strain), we compute image features for all of its 24 crops and concatenate them into a one-dimensional vector, which is considered as the final image feature vector of each strain profile. Thus, the euclidean distance between these strain profiles can be calculated to find nearest neighbors. In this way, the stress-strain curve up to large-reload (i.e., 1% reloading strain) can be obtained by averaging the stress-strain curves of nearest neighbors.

**Figure 5:**
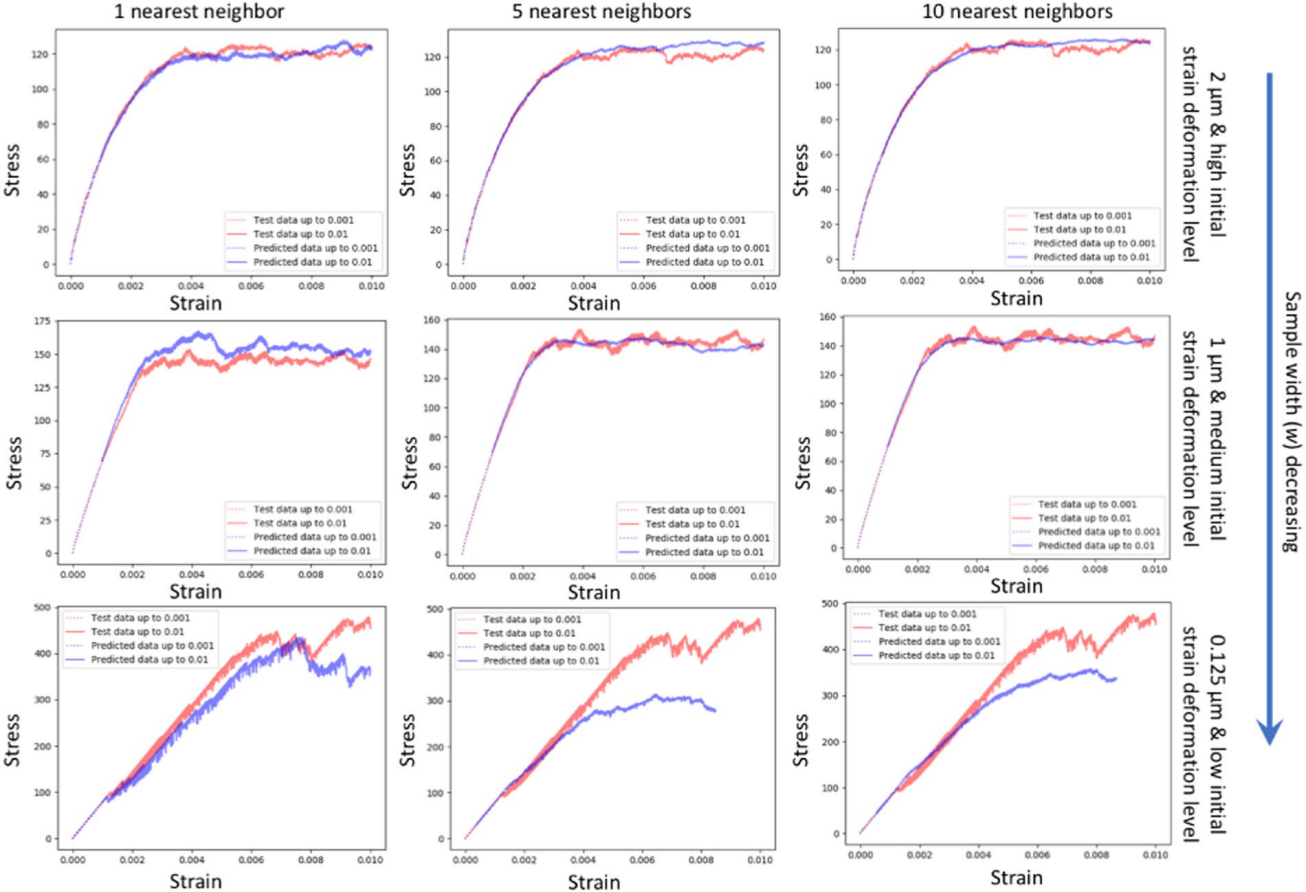
The comparison of true stress-strain curve and averaged stress-strain curves of nearest neighbors for strain profiles with different widths and different prior deformation levels. Each row from top to bottom presents the plots of strain profiles with 2 *μm* width and high initial strain deformation level, 1 *μm* width and medium initial strain deformation level, and 0.125 *μm* width and low initial strain deformation level, respectively. Each column from left to right shows the averaged stress-strain curves of 1, 5 and 10 nearest neighbors for the strain profile of interest, respectively. It shows that stochasticity and size effect increases as sample width decreases.

Figure 5 shows the comparison of true stress-strain curve and averaged stress-strain curves of nearest neighbors for strain profiles with different widths and different prior deformation levels. Similar to the prediction of initial strain deformation level, we can observe that the predicted stress-strain curves can accurately capture the yield points as well as match true stress-strain curve well when the sample width is large (i.e., 2 *μm* and 1 *μm*). However, when the sample width decreases, the error becomes significant. In addition, the noise level of predicted stress-strain curve decreases (i.e., the curve becomes smoother) by averaging stress-strain curves of more nearest neighbors.

### Visualization of the deep learning model

Although deep learning has shown its striking learning capability in many research tasks, it is usually considered as a black box. Thus, it is interesting to see what the deep learning model has learned. In this section, we visualize the filters in convolutional layers and plot saliency maps using the proposed deep learning model to try to understand what it has learned.

**Figure 6:**
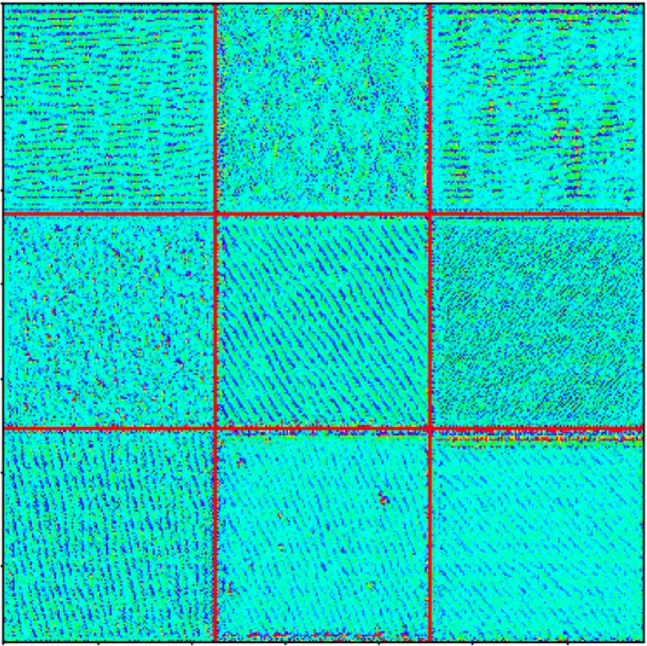
Visualization of nine filters of the last convolutional layer in the second residual module of the proposed model.

First, we visualize what the filters in covolutional layers learn. Particularly, the filters of the last convolutional layer in the second residual module of the proposed model are visualized. To do this, we start from a gray scale image with random noise as values of pixels, then we compute the gradient of this image with respect to the “loss function”, which is defined to maximize the activations of the filter in the selected layer. For each filter in the selected layer, we run gradient ascent (as opposed to descent, since in this case we are interested in maximizing the loss function instead of minimizing) for 100 backpropagation iterations to update its input image. Finally, the images from the corresponding filters that have the highest loss are plotted in Fig. 6. We can observe that the filters can learn lines with various characteristics, such as widths and orientations. As the main difference between the strain profiles is the characteristics of the dislocation lines in the images (e.g., the number, width and orientation), the higher level layers can utilize these learned features to identity the dislocation lines in the strain profile so that the model can accurately classify them.

**Figure 7:**
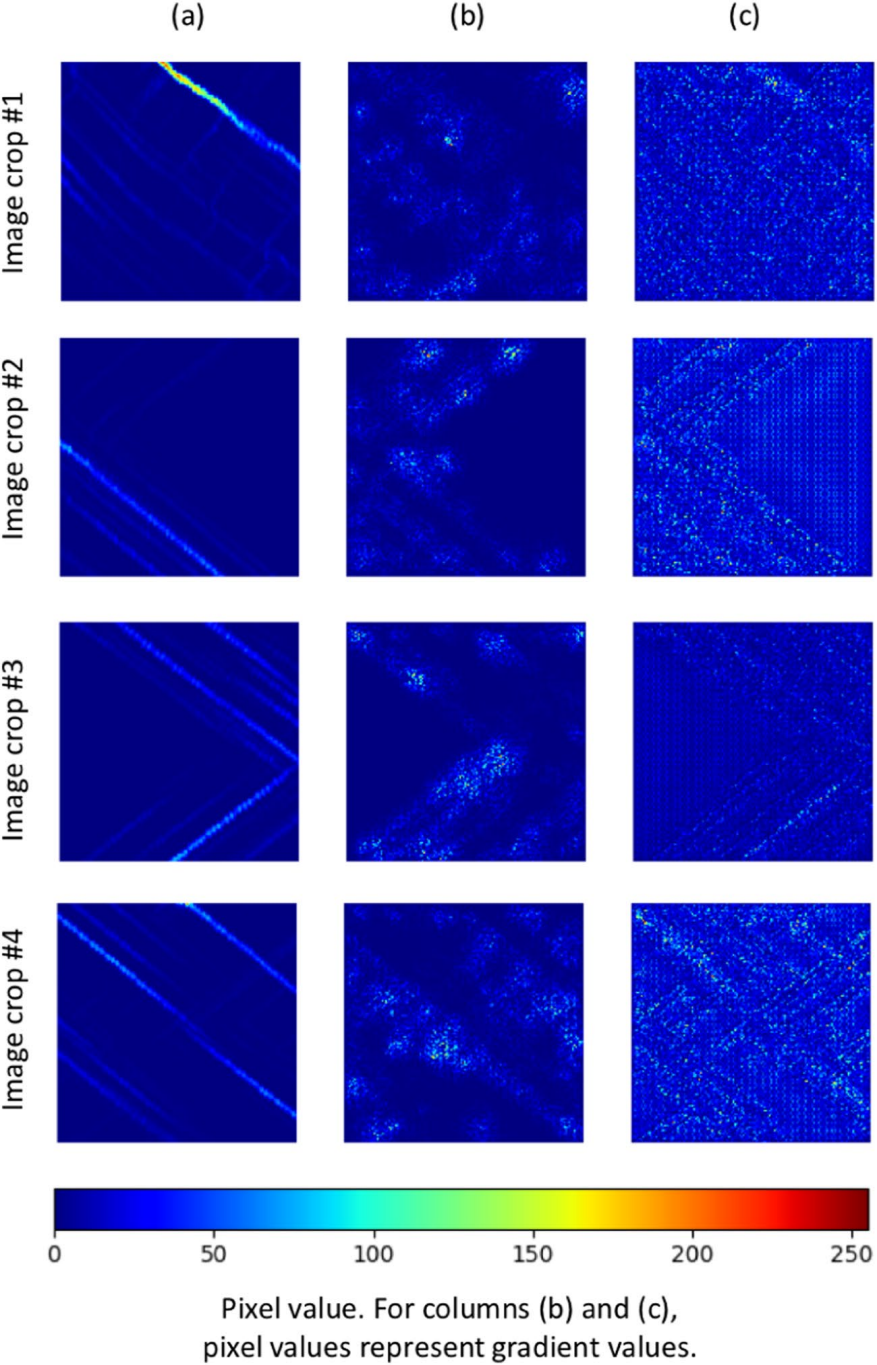
Visualization of saliency maps of five image crops of strain profiles. (**a**) original image crop. (**b**) saliency map without back propagation modifier. (**c**) saliency map with ReLU as back propagation modifier.

Next, saliency maps^53^ are visualized to determine which aspect of the proposed model is the most important to obtain accurate predictions. To plot the saliency map, we compute the gradient of the initial strain deformation level with respect of the input image. This gradient shows how the probability of prediction changes with respect to a small change in the input image, which intuitively highlights the salient image regions that dominate the prediction. Figure 7 shows saliency maps of four image crops of strain profiles. Particularly, the first column shows the original image crop, the second column presents the saliency maps without back propagation modifier, and the third column illustrates the saliency maps with ReLU as back propagation modifier, which means only the positive gradients can be back propagated through the network. The dislocation lines in the strain profiles are crucial to identify the initial strain deformation level, and the saliency maps show that the gradients are large around the dislocation lines, which means that the proposed model pays more attention to those regions to capture salient information and make accurate predictions.

**Figure 8:**
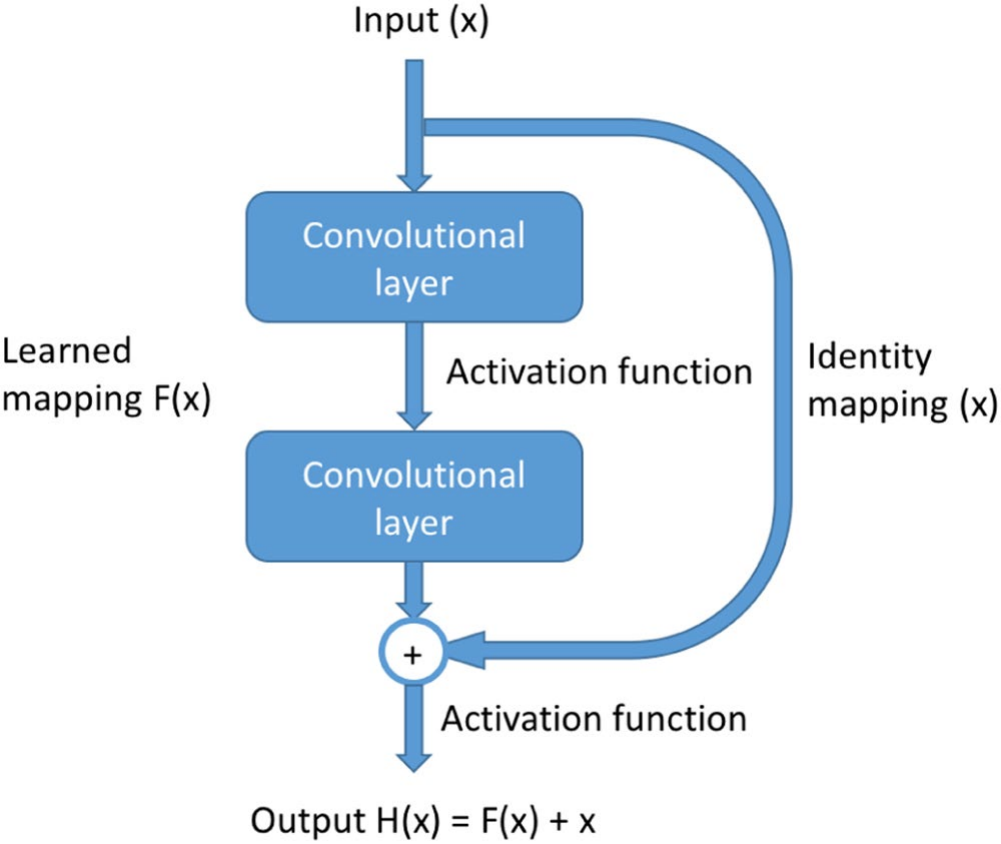
Illustration of Residual module.

## Discussion

In this work, we develop a deep learning network combined with the state-of-the-art techniques, such as residual module and batch normalization, to predict the initial strain deformation level based on strain profiles. The results show that the proposed model can achieve 92.48% classification accuracy, and the classification accuracy is further improved by an ensemble of three different trained deep learning models. The results for samples of different subsets matches domain knowledge that the initial strain deformation level is more difficult to predict when sample width is too small. More importantly, the results show that the output of second to last layer of the proposed model is a good representation of the strain profiles, which can be used to compute nearest neighbors and predict stress-strain curve for the sample of interest. In addition, by visualizing the filters in the convolutional layer and plotting the saliency maps, we can observe that the proposed model can identify dislocation lines and capture the salient regions of the strain profiles, which results in the accurate predictions.

Traditional research methods in materials science (i.e., experiment and simulation) have obvious drawbacks. Experiments are the most reliable method, but are extremely expensive in terms of cost and time. More importantly, some experiments might change the mechanical property of materials or even destroy them, such as tensile test. On the other hand, although simulations are never able to incorporate all the variables and constraints that are associated with an actual experiment, it is a faster way that tries to reproduce the experiment process. However, it might take hours or days to finish a complicated computation process, which hinders its use to create a large dataset. As large reliable data are available nowadays^54,55^, deep learning approach shows its superiority to traditional computational methods. With striking learning capability and flexible architecture, deep learning models can usually provide accurate predictions in an efficient manner, which can be used to augment traditional computational methods.

**Figure 9:**
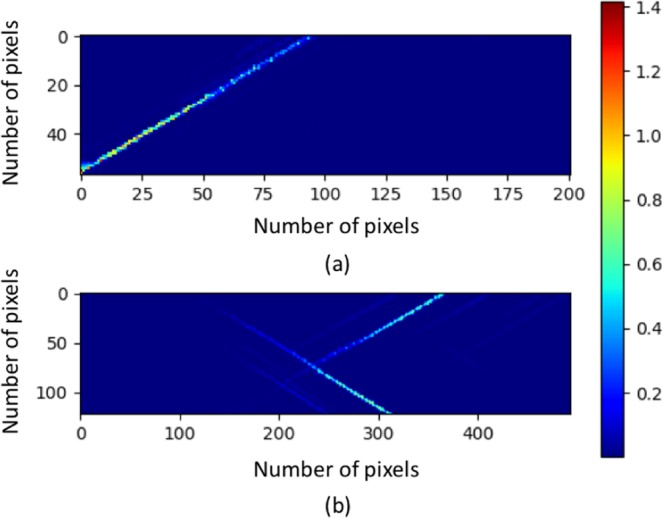
Examples of strain profiles. (**a**) sample of a width of 0.125 *μm* with large-reload and one slip system. (**b**) sample of a width of 0.25 *μm* with large-reload and two slip system.

From data mining point of view, the advantages of the proposed model are threefold compared to the benchmark machine learning method: (1) Better accuracy: the classification accuracy of the proposed method significantly outperforms the benchmark method (2) Good representation: The output of second to last layer could be a good representation that accurately characterizes the strain profiles. (3) Interpretation: The saliency maps and convolutional layer filters visualization indicate that the proposed model indeed captures the important characteristics of the strain profile, which makes the results more trustable. However, there are also limitations for the proposed model: (1) The current model treats the problem as a classification problem. Ideally, a regression model should be developed that can predict any initial strain deformation level given a strain profile. However, the lack of data for other initial strain deformation levels hinders the development of the regression model. Although the data collection takes a significant amount of time and effort, once the dataset is established it would become an important resource to accelerate the crystal plasticity research, building upon the current classification model. (2) Image cropping is used as a preprocessing step to augment the dataset. However, as shown in Fig. 9, most of the dislocation lines are located around the diagonal of the strain profile. Thus, image cropping might produce some crops that contain small regions with no dislocation lines, and such crops might significantly confuse the model in both training and testing time. To solve this problem, a domain knowledge based filter could possibly be designed to remove crops without important dislocation information before feeding them into the model.

From the perspective of materials science, the impact and applications of the proposed method are also significant. Prior deformation strain is difficult to detect from a visual inspection of the materials. The proposed method provides an accurate and efficient way to identify the initial strain deformation level, which is used to further predict the stress-strain curve of the sample. Thus, the proposed method could be used in industry, such as automotive manufacturing companies. More specifically, the proposed method could be used to do a prescreening on the engineering parts, and a thorough examination could be done on the suspected parts, which could significantly reduce cost and increase production efficiency. In addition, the proposed methodology could be easily extended to other materials systems. On the one hand, because the proposed method only takes strain profiles as inputs without any ad hoc assumptions about the materials, the methodology could be easily extended to solve similar research tasks on similar inputs. On the other hand, although the results of this work is based on strain profile data (i.e. 2D case), it could be easily extended to heterogeneous materials (i.e. 3D cases where strain is heterogeneous through the thickness), because CNN has shown its ability to provide accurate predictions on both 2D and 3D materials data^38,42,56^. To extend the proposed methodology on heterogeneous materials, the input layer of CNN architecture needs to be changed. More specifically, the images of different consequent layers or representative layers of heterogeneous materials would be stacked together to form a 3D image, which is considered as a 2D image with multiple channels in deep learning. Then, it would be fed into CNN so that CNN can learn features across different channels toward accurate predictions^57–59^. Note that architecture and hyperparameter settings might need to be tuned for different research tasks.

There are two interesting topics can be further investigated in the future work. First, this work is focused on uniaxial compression studies. We consider extensive applications of compression/decompression on samples in order to generate physically sensible dislocation microstructures. The application of sequential compression/decompression and the consequent effect on sample dislocation densities can be thought as corresponding to a strain path effect investigation, in consistency to observed compression/tension anisotropy in crystalline nanopillars^6^. Strain path changes in directions other than the chosen uniaxial loading one can be further investigated in the future. Second, since the proposed method could be extended to 3D materials data, the study of crystal plasticity on three dimensional heterogeneous materials is an interesting topic of investigation for future works.

## Methods

The background about digital image correlation and discrete dislocation dynamics, and deep residual learning is introduced in section 4.1 and 4.2, respectively. Then, we describe the dataset and data preprocessing in section 4.3 and 4.4. Finally, the deep residual learning model is proposed in section 4.5.

### Digital image correlation and discrete dislocation dynamics

Digital image correlation (DIC) is an optical method using tracking and image registration techniques to accurately measure the changes in a two-dimension or three-dimension image, and it is widely used to map deformation in macroscopic mechanical testing^60,61^. In DIC, the gray scale images of a sample at different deformation stages are compared to calculate displacement and strain using a correlation algorithm. In this work, the DIC is emulated using discrete dislocation dynamics (DDD) simulation. In DDD, dislocation lines are represented explicitly, where each dislocation line is considered as an elastic inclusion embedded in an elastic medium. The interacting dislocations, under an external loading condition, are simulated using elastic property of the material. More specifically, as in^13^, material sample is stress free without mobile dislocations at the beginning of the DDD simulation. In this work, we neglect the possibility of climb and only consider glide of dislocations. Thus, the motion of dislocations is determined by Peach-Koehler force in the slip direction. Once nucleated, dislocations can either exit the sample through the traction-free sides or become pinned at the obstacle. If dislocations approach the physical boundary of the material sample, a geometric step is created on the surface along the slip direction. After the material sample has been strained and relaxed, it is then subjected to a subsequent “testing” deformation so that strain field can be measured.

### Deep residual learning

Though the concept of convolutional neural network (CNN) has been well-known for a long time, it did not get much recognition until AlexNet^62^ was introduced, which won the 2012 ImageNet ILSVRC challenge^63^, and significantly outperformed the runner-up. Many new CNN architectures and related techniques, such as VGGNet^64^ and GoogLeNet^52^, have been developed since then to improve the model performance and solve various tasks in computer vision. However, when the architecture becomes deeper and deeper (i.e., more hidden layers), a degradation problem has been exposed where the accuracy decreases with increasing the depth of the deep learning model. In order to solve this problem, He *et al*.^23^ proposed a residual network to take advantage of the high learning capability of a deeper model and avoid the degradation problem. Figure 8 is an illustration of residual module. In contrast to conventional CNN where convolutional layers are trained to directly learn the desired underlying mapping, the convolutional layers in residual module learn a residual mapping. To achieve this, a shortcut connection^65^ is introduced to perform identity mapping where the input *x* is added to the output of stacked convolutional layers. Thus, instead of learning underlying mapping *H*(*x*), the stacked convolutional layers are used to learn the residual mapping *F*(*x*) = *H*(*x*) − *x*. In this way, if the identity mapping is already optimal and the stacked convolutional layers cannot learn more salient information, it can push the residual mapping to zero so as to avoid the degradation problem. The residual module is shown to be easier to optimize so that deeper architecture can be developed.

### Dataset

The dataset is generated by 2D DDD simulations. In this simulation, a sample is loaded to a high strain deformation level and then unloaded. After that, the sample is reloaded to a testing strain, and we can obtain the strain profile at the testing reloading strain. Figure 9 shows two examples of strain profiles. The dataset has 3648 strain profiles and includes three variables, which are sample width, testing reloading strain and slip type of material system. More specifically, there are six different sample widths (i.e., 2 *μm*, 1 *μm*, 0.5 *μm*, 0.25 *μm*, 0.125 *μm* and 0.0625 *μm*), two testing reloading strains (i.e., 0.1% and 1.0%, which are referred as small-reload and large-reload in the rest of the paper), and two slip types (i.e., one slip system and two slip system). Thus, there are 24 subsets of data in total, and the number of data points in each subset is listed in Table 3. Each strain profile can be considered as a two-dimensional one-channel image, and the pixel values are continuous values representing the local strains. However, the image size of the strain profiles are different for different sample width (increasing as a factor of the sample width). Meanwhile, the image size of strain profiles with the same sample width can also be slightly different. For example, the image size of a sample with a width of 0.125 *μm* is around 60 × 200 (see Fig. 9(a)), while the image size of a sample with a width of 0.25 *μm* is around 120 × 500 (see Fig. 9(b)). In order to cover the representations from all the subsets, we randomly select around 25% data from each subset as testing set. For rest of the data, around 82% is used for training, and the remaining for validation. In other words, the dataset is split into three sets where training set has 2253 data points, validation set includes 504 data points and testing set contains 891 data points. The response of each sample to be learnt is its initial strain deformation level of low medium, or high (i.e., 0.1%, 1.0% and 10.0%), which means this is a three-class classification problem.

**Table 3:** Number of Data Points for Each Subset in Crystal Plasticity Dataset.

Width (*μm*)	2	1	0.5	0.25	0.125	0.0625
Small-reload & two slip	154	156	154	156	155	156
Small-reload & one slip	147	147	149	150	150	150
large-reload & two slip	151	156	154	156	155	156
large-reload & one slip	150	147	149	150	150	150

### Data preprocessing

**Figure 10:**
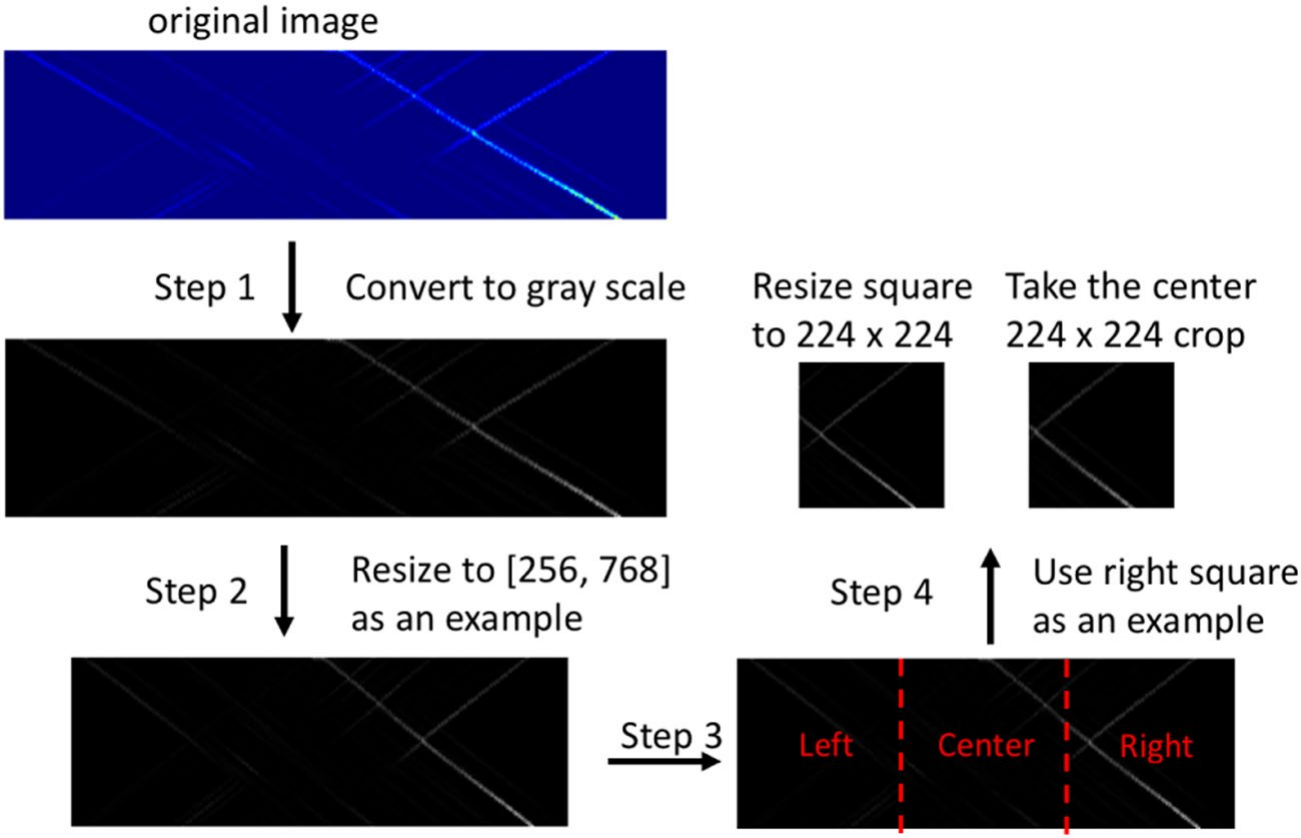
Illustration of image preprocessing. As an example, the image is resized to [256, 768] in step 2 and right square is used in step 4 in this figure.

Because the dataset is relatively small to train a deep learning model and the image size is varied, an four-step data preprocessing approach is used to augment the dataset and convert it into a format that is suitable for training a deep learning model. Figure 10 illustrates the image preprocessing steps.

- Image (i.e., strain profile) is converted to gray scale image, which means the values of pixels in the image are rescaled to [0, 255].
- Converted image is resized to four scales where the shorter dimensions are 256, 288, 320 and 352, with an aspect ratio of 1:3.
- The left, middle and right squares are cropped for each resized image.
- The center 224 × 224 crop as well as the square resized to 224 × 224 are taken for each square.

Thus, each image can have 24 crops, which means the dataset is enlarged 24 times. Note that during testing, the softmax probabilities of all the crops of a test image are summed up, and the class with the highest probability is declared as the final prediction.

### The proposed deep learning model

**Figure 11:**
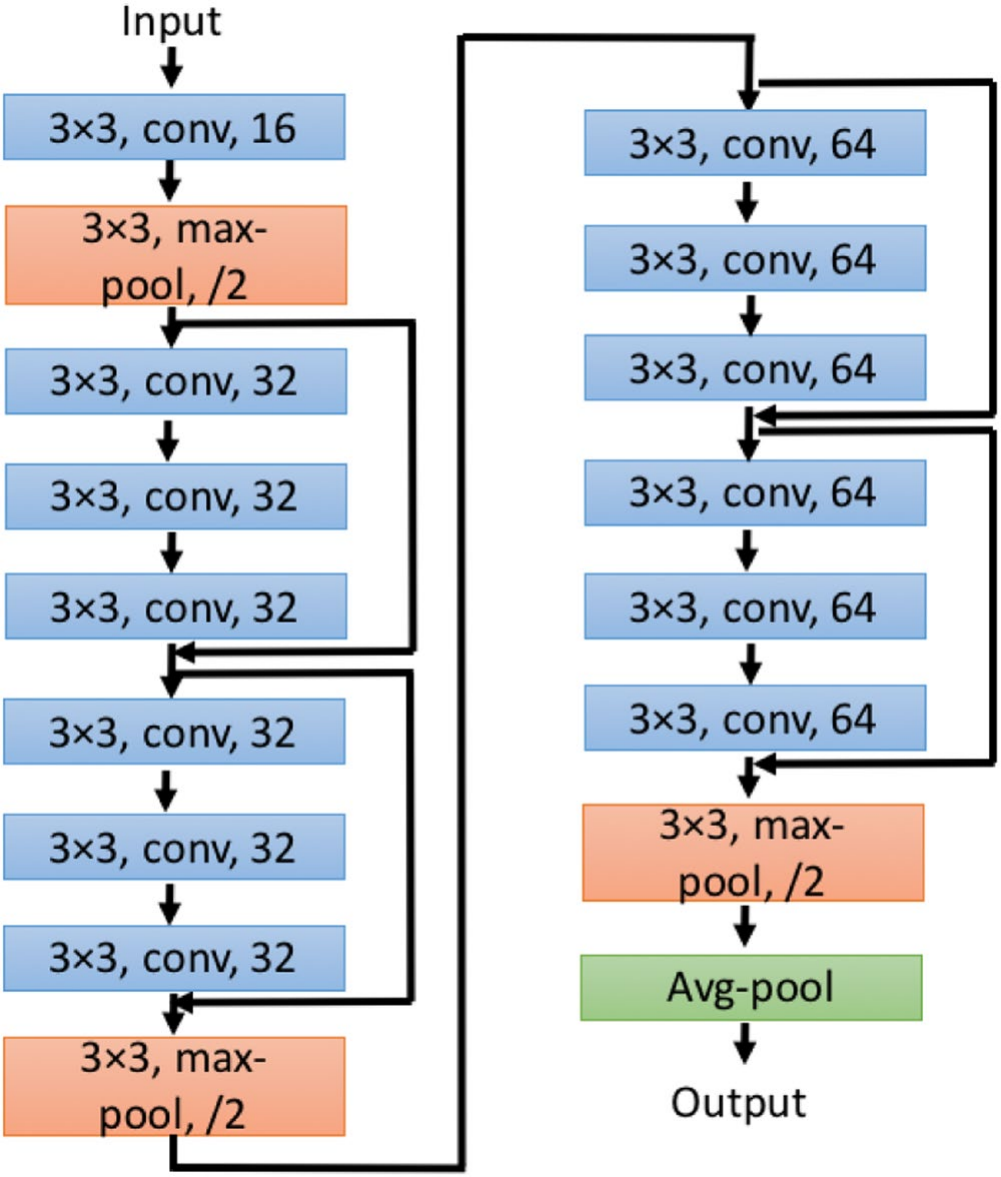
The architecture of the proposed CNN. “3 × 3, conv, 16” indicates that the convolutional layer in this convolutional block has 16 3 × 3 filters. “3 × 3, max-pool, /2” indicates that the max pooling layer is 3 × 3 with stride 2. “Avg-pool” denotes a global average pooling layer.

A deep residual network with state-of-the-art techniques, such as residual module^23^ and batch normalization^66^, is developed to train the predictive model. Let “conv” denote a convolutional block (i.e. a convolutional layer followed by a batch normalization layer and an activation function layer), “pool” a max pooling layer, “Res” a residual module and “avgpool” a global average pooling layer^67^. The architecture of the proposed model can be described as *input* − *conv16* − *pool* − (*Res*32) × 2 − *pool* − (*Res*64) × 2 − *pool* − *avgpool* − *output* and it is illustrated in Fig. 11. More specifically, the proposed model takes the preprocessed images as input, followed by a convolutional block and its convolutional layer has 16 filters, and then followed by a max pooling layer. Then, there are two residual modules, and each residual module includes three convolutional blocks and the convolutional layer of each convolutional block has 32 filters. The convolutional layer for the identity mapping in these two residual modules has 32 1 × 1 filters. Then they are followed by a max pooling layer. After that, another two residual modules are attached, and each one has three convolutional blocks and the convolutional layer of each convolutional block has 64 filters. The convolutional layer for the identity mapping in the two residual modules has 64 1 × 1 filters. Then a max pooling layer is applied. Finally, a global average pooling layer is attached and the output layer is used to produce the final prediction. Unless otherwise specified, the size of all the filters in convolutional layer are 3 × 3, and all the max pooling layers are 3 × 3 with stride 2. Rectified Linear Unit (ReLU)^68^ is used as the activation function for all the convolutional blocks and a softmax activation function is used for the output layer. In order to avoid overfitting, early stopping is applied where the training process is terminated if the value of loss function on validation set is not improved for 20 epochs. In addition, Adam^69^ with learning rate as 0.001, *β*_1_ as 0.9 and *β*_2_ as 0.999 is used as optimizer. Each batch includes 72 images for training.

## Data Availability

The data that support the findings of this study are available from corresponding authors on reasonable request.

## Code availability

The trained model and weights are available at https://github.com/zyz293/plasticity-DL.
